# Modification and Aging Mechanism of Crumb Rubber Modified Asphalt Based on Molecular Dynamics Simulation

**DOI:** 10.3390/ma18010197

**Published:** 2025-01-05

**Authors:** Jian Li, Liang He

**Affiliations:** 1 School of Civil Engineering, Chongqing Jiaotong University, Chongqing 400074, China; lianghe@cqjtu.edu.cn; 2 Guangxi Transport Vocational and Technical College, Nanning 530023, China

**Keywords:** crumb rubber modified asphalt (CRMA), molecular dynamics (MD), modification mechanism, aging, interfacial interaction

## Abstract

Asphalt modified with treated waste tires has good environmental protection and application value. However, the nano-modification mechanism of crumb rubber (CR) with asphalt is still unclear. This research investigates the mechanism, aging, and interfacial interaction with the aggregate of CR modification asphalt (CRMA). The base asphalt and CRMA (original and aged) and two typical aggregate models were constructed. The accuracy of the model was verified through multiple indicators. The effects of CR and aging on the physical properties (density, compatibility, and diffusion coefficient), mechanical properties, component interaction behavior, and interfacial interactions with aggregates of CRMA were systematically analyzed. The results showed that the CR reduced the diffusion coefficient of asphalt by about 31%. The CR inhibited the movement of the components of asphalt (especially saturate and aromatic), which significantly improved the mechanical properties of asphalt. The compatibility between asphalt and CR significantly deteriorated after aging. The difference in the solubility parameter was about four times that before aging. It is instructive for the regeneration of CRMA. Aging led to a decrease in the shear modulus and Young’s modulus of both base asphalt and CRMA, which verified and quantified the adverse effects of aging on the mechanical properties. Comparing the two aggregates, CaCO_3_ had a greater adhesion with asphalt than SiO_2_. The difference ranged from 22.5% to 39.9%, which quantified the difference in the adhesion properties of acid base aggregates with asphalt. This study can provide theoretical guidance for the modification and application of CRMA.

## 1. Introduction

Mass tire waste has been generated with the rapid rise of vehicle ownership, causing ecological and environmental problems [1,2]. How to effectively reuse these waste tires has become one of the critical issues in building a resource-saving and environmentally friendly society. In road engineering, crumb-rubber-modified asphalt (CRMA) can be prepared by grinding waste rubber tires into crumb rubber (CR) [3,4,5]. Adding CR can improve the asphalt performance and reduce road noise [6,7,8,9]. It has gradually become widely used in road engineering in recent years.

Many researchers have studied CRMA, especially its modification, aging, and asphalt–aggregate adhesion properties, which are critical to the performance and service life of asphalt pavement. For example, in terms of modified modification properties, Jeong et al. [10] tested the modification mechanism and rheological properties of CRMA using a dynamic shear rheometer and chromatographic gel analysis. The results indicated that the reaction time and the temperature had the most significant effect on CRMA. Peng et al. [11] systematically evaluated the improving effect of CRMA after improving the preparation process, including the rheological properties, hydrophilic and hydrophobic properties. Su et al. [12] evaluated the effect of CR and asphalt through solvent elution and microscopic tests. Chen and Wang et al. [13,14] used a bending beam rheometer and differential scanning calorimeter to determine the effect of aging on the low-temperature performance and fatigue resistance of CRMA. It showed that the CRMA had better thermal stability and low-temperature performance. Shen et al. [15] investigated the adhesion mechanism between CRMA and a steel slag aggregate by using the pull-out test and SEM microscopic observation. However, the conclusions reached have usually been limited to the macro level due to the limitations of the test equipment and test methods, even if microscopic evaluation techniques such as FTIR and SEM are used. Due to the complex composition of CR, its distribution and role in asphalt are difficult to observe visually.

Molecular dynamics (MD) is a computerized virtual simulation method. It can simulate the motion of molecular systems through Newtonian mechanics, effectively making up for the lack of macroscopic tests [16,17,18]. In recent years, MD has been widely used in asphalt research. Hu et al. [19] used MD to study the interfacial properties of CRMA from the perspective of thermodynamic parameters. Xu et al. [20] used MD to simulate the oxidative aging effect of asphalt. They investigated the effect of aging on the self-healing ability of asphalt and its susceptibility to moisture damage. Wang et al. [21] and Qu et al. [22] studied the aging effect on the physical properties of asphalt using MD by establishing a molecular model of six different aging states. Chen et al. [23] and Gong et al. [24] studied the effect of temperature and water on the adhesion properties of the asphalt–aggregate interface using MD. The above studies were based on MD techniques and investigated the action mechanisms of different aspects of CRMA. However, the CRMA models used above varied greatly due to the differences in CR’s sources and preparation processes. This led to unsystematic findings in different literature. Some even have opposite conclusions. In a comprehensive analysis, the existing research on CRMA still has the following research gaps:

(1) Existing experimental studies have not explained the mechanism of modification, aging, and interfacial interaction with CRMA aggregates. This was also confirmed by the fact that there were still significant limitations in promoting the use of CRMA [1,11].
(2) In the MD study, there is still a need to deepen the research further in terms of the accuracy of CRMA model construction and the comprehensiveness of the mechanism of action study.

This study aims to analyze the modification, aging, and interfacial interaction with the aggregate mechanism of CRMA using the MD method, expecting to guide the production of CRMA modification. Firstly, an optimized model of CR was constructed, and original and aged base asphalt and CRMA models were built. Through the model validation, physical properties, mechanical properties, diffusion coefficient, and binding energy, the modification, aging, component action behavior, and the influence of the interface on the aggregate mechanism of CRMA were evaluated. The research results can provide theoretical guidance for the modification and engineering application of CRMA.

## 2. Modeling and Simulation Methods

### 2.1. Modeling of Base Asphalt Models

Li and Greenfield [25] proposed a four-component asphalt model with twelve molecules. This model can more accurately simulate the structure of asphalt, and is currently the most commonly used model [26,27,28]. Therefore, this study chose the twelve-molecule asphalt model to construct the original base asphalt model.

**Figure 1:**
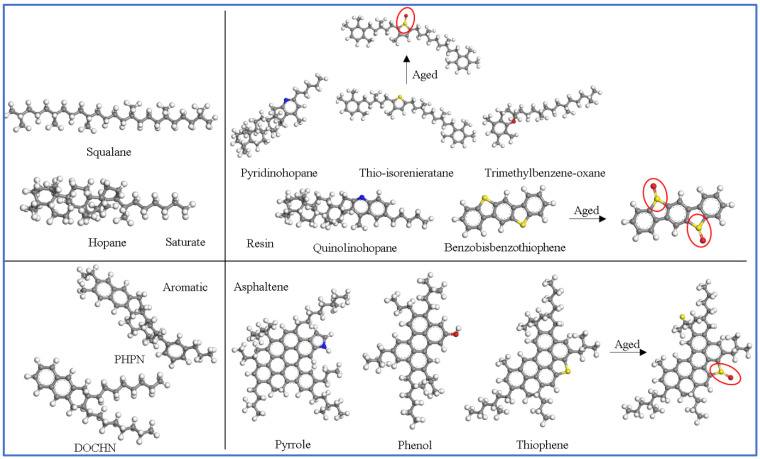
Molecular structure of the original and aged base asphalt model.

The molecular structure of asphalt and the proportion of components were changed after aging. So, the aged base asphalt model was constructed by adjusting some structures and changing the proportion of fractions [29]. The molecular structure of the original and aged base asphalt is shown in Figure 1 (marked sections indicate molecules that have changed with aging). The molecular composition is in Table 1. The original and aged base asphalt models were constructed based on the number of molecules in Table 1.

### 2.2. Modeling of Improved CRMA Models

**Figure 2:**
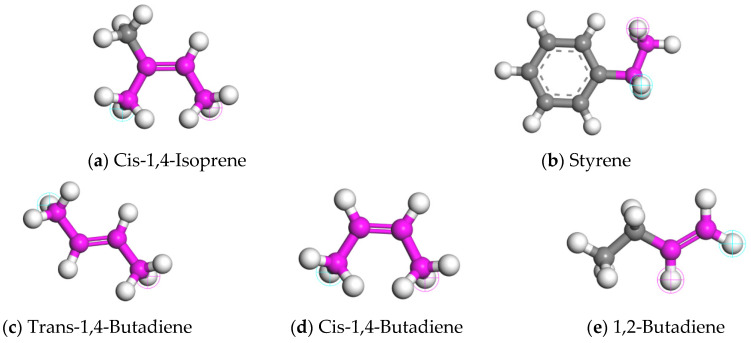
Molecular structures of NR and SBR synthetic monomers.

The CR used in CRMA is mainly derived from waste tires. However, the selection of molecular models for CR is not uniform in the current studies. Significant differences existed in the type, proportion, and polymerization degree of the constituent molecules [21,30,31,32,33,34,35,36,37]. Natural rubber (NR) and styrene butadiene rubber (SBR) were selected as the main components to construct a more accurate CR model. The mass ratio of NR to SBR is 7:3 [30,31]. NR is a homopolymer, and the synthetic monomer is cis-1,4-isoprene. SBR is a random copolymer, and the synthesis monomers are styrene and butadiene. Butadiene contains different structures, including trans-1,4-butadiene, cis-1,4-butadiene, 1,2-butadiene, etc. The molecular structures of five synthetic monomers are shown in Figure 2. The repeat unit content of each structure of SBR is shown in Table 2.

**Figure 3:**
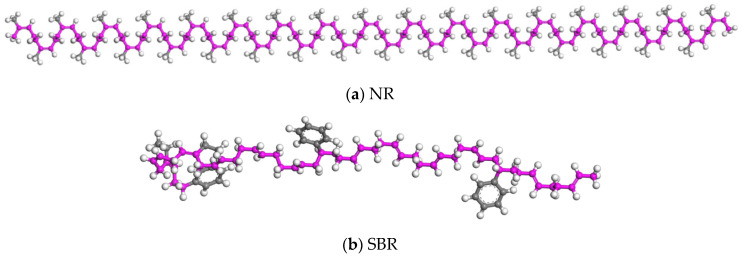
Rubber single-chain model.

The dosage of CR was 20 wt% in CRMA. Based on the mass ratio of NR to SBR, the molecular numbers of the two rubbers can be calculated. The single chain of the rubber model could also be constructed (as shown in Figure 3).

**Figure 4:**
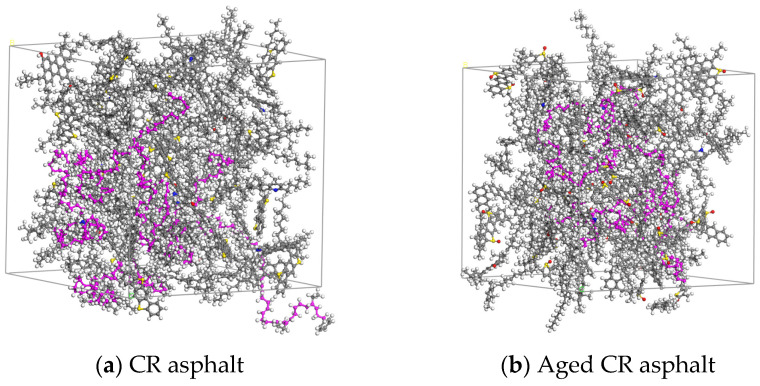
Original and aged CRMA models.

The CR (the purple part of the figure), original, or aged base asphalt models were scaled to construct CRMA models. The original and aged CRMA models are shown in Figure 4.

### 2.3. MD Simulation Details

All MD simulations were carried out using Materials Studio software. The Condensed-phase Optimized Molecular Potential for Atomistic Simulation Studies (COMPASS) II force field was employed. The asphalt models constructed by the above method were not yet stable enough. Therefore, the models were subjected to 10 simulated annealing operations using the Forcite module. The temperature range was 298–1000 K under a barometric pressure of 1 standard atmospheric pressure. The tandem combination of the Steepest descent method, the ABNR method, and the Quasi-Newton method was chosen as the optimization algorithm for the geometric optimization, with a maximum number of iterations of 5000. Finally, the dynamic equilibrium of the NPT system was carried out at 298 K. Nose was selected for the thermostat and Berendsen was selected for the constant voltage, and the total simulation time was set to 1000 ps, with a time step of 1 fs. The equilibrium model with a stable volume and energy was obtained, and the trajectory file equilibrium structure was taken for the subsequent parameter calculations.

### 2.4. Modeling of Asphalt–Aggregate Interface Models

#### 2.4.1. Aggregate Models

Two aggregate types, limestone and granite, with significant differences in acidity and alkalinity, were selected for analysis. Granite has quartz as its main mineral constituent, and limestone has calcite as its main mineral constituent. Therefore, quartz (SiO_2_) and calcite (CaCO_3_) were selected as representative aggregates. Taking SiO_2_ as an example, the SiO_2_ cell model was imported from the Materials Studio (MS) software. The (0 0 1) surface of the cell was cut using the Build-Surfaces-Cleave Surface command (the (0 1 8) surface of the CaCO_3_ cell was cut). The model was orthogonalized using the Build-Symmetry-Redefine Lattice command. The SiO_2_ surface was then hydroxylated. A vacuum layer was added using the Build-Crystals-Build Vacuum Slab Crystal command. Finally, the supercell model was extended using the Build-Symmetry-Supercell command to make the dimensions as close as possible to the asphalt model dimensions.

#### 2.4.2. Asphalt–Aggregate Interface Models

**Figure 5:**
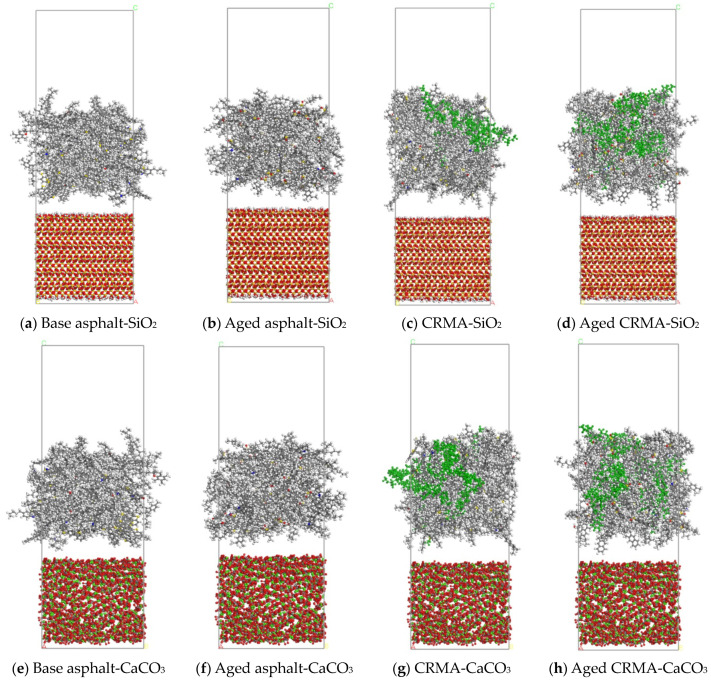
Asphalt–aggregate interface model.

The Build Layer function was used to build the asphalt–aggregate interface models with optimized asphalt and aggregate models. A vacuum layer of 30 Å was added above to avoid the influence of periodic boundary effects in the Z direction. The lower part of the aggregate model was fixed to simulate the actual situation better. The interface model is shown in Figure 5.

## 3. Results and Discussion

### 3.1. Model Validation

The optimized base asphalt model was used to calculate the density, glass transition temperature (*T_g_*), cohesive energy density (CED,) and solubility parameter (*S_p_*). It was verified that the properties of the asphalt matched the actual indexes.

#### 3.1.1. Density

**Figure 6:**
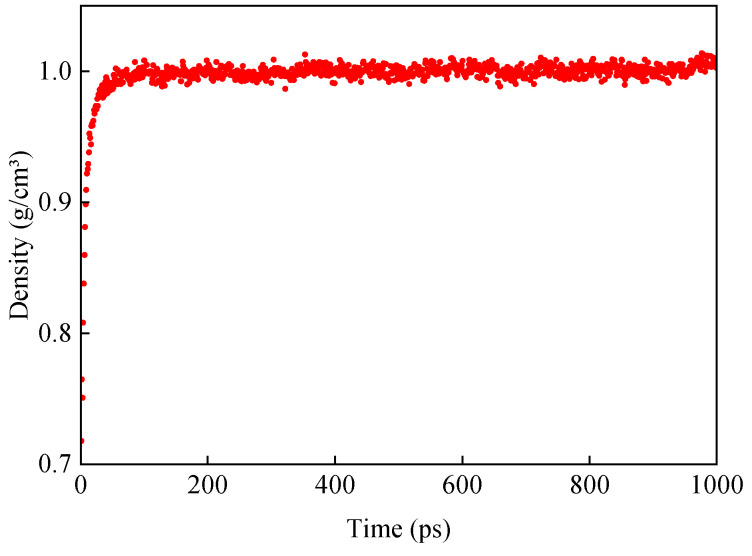
Variation in density of original base asphalt.

The density of the original base asphalt model was obtained after the NPT system was subjected to dynamic equilibrium at 298 K for 1000 ps. The mean density of the last 100 ps was taken as the representative density of the model. The density of the base asphalt model was calculated to be 1.003 g/cm^3^, as shown in Figure 6. The measured density of base asphalt generally ranges from 1.01 to 1.04 g/cm^3^ [38]. This indicated that the model construction and parameterization were reasonable for subsequent MD analysis [39].

#### 3.1.2. *T_g_*

Asphalt is a viscoelastic material. Depending on the molecular motion, the rheological behavior can be classified into different temperature-dependent states: viscous, viscoelastic, and glassy. The *T_g_* of asphalt is a characteristic temperature at which the glassy state of asphalt can be reversibly transformed to a rubbery viscoelastic state and vice versa. The physical properties, such as the specific volume, modulus, and specific heat, will change dramatically at this transition point. According to the free volume theory, the glass transition temperature can be defined as the intersection of the thermodynamic equilibrium lines in the rubber-like and glass-like regions, respectively. Molecular dynamics simulations are often used to calculate the glass transition temperature of a material according to the change in the material properties (density, specific volume, etc.) with temperature, and the specific volume is calculated as shown in Equation (1).

(1) $$V_{\mathrm{specific}}=\frac{1}{\rho }$$

where *V_specific_* is the specific volume and *ρ* is the model density.

**Figure 7:**
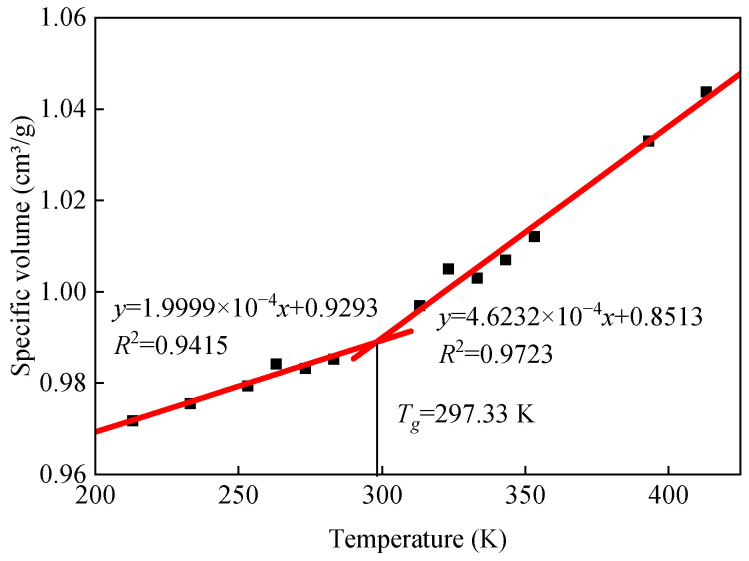
Variation curve of base asphalt specific volume with temperature.

The results of specific volume calculations for the base asphalt model at different temperatures are shown in Figure 7. A further analysis of the data in Figure 7 yields a base asphalt model glass transition temperature of 297.33 K, which agrees with the simulation results in the literature (278.66 K) [40].

The CED indicates the energy required to overcome the intermolecular forces to vaporize the condensate per unit volume. The CED reflects the magnitude of the intermolecular forces in the system. The solubility parameter is a physical constant that measures the compatibility of polymer materials, and its physical meaning is the open square of the CED of the material. The CED and *S_p_* were calculated by Equations (2)–(4).

(2) $$E_{\mathrm{coh}}=-E_{\mathrm{inter}}=E_{\mathrm{intra}}-E_{total}$$

(3) $$CED=\frac{E_{coh}}{V}$$

(4) $$S_{p}=\sqrt{CED}$$

where *E_coh_* is the cohesion energy, *E_inter_* is the intermolecular interaction energy, *E_intra_* is the intramolecular interaction energy, *E_total_* is the total system interaction energy, and *V* is the model volume.

The CED of the original base asphalt model was calculated using the Forcite module at 3.17 × 10^8^ J/m^3^, and the solubility parameter was 18.214 (J/m^3^)^1/2^. The comparison showed that the simulation results were consistent with the experimental values (CED of 3.31~3.73 × 10^8^ J/m^3^ and *S_p_* of 18.19–19.31 (J/m^3^)^1/2^) in the literature [41]. Therefore, the selection of the asphalt component model and the analytical parameters set, such as the force field, were reasonable.

### 3.2. Physical Properties

#### 3.2.1. Density

**Figure 8:**
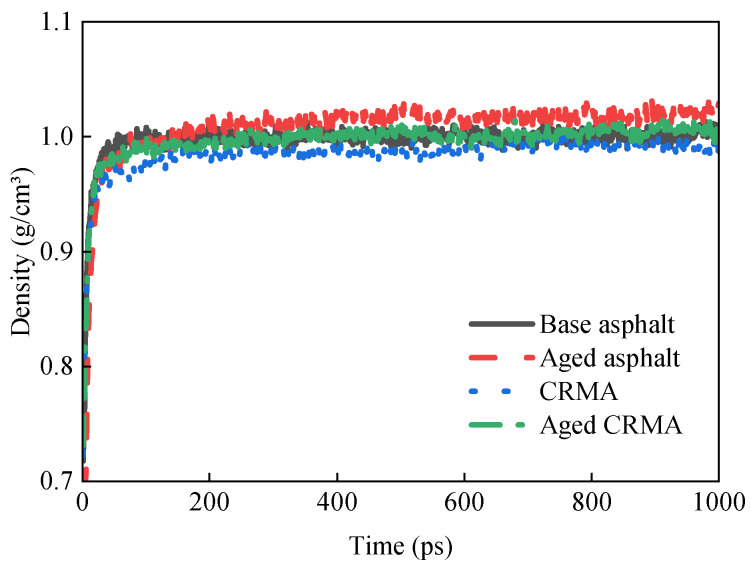
Variation in density of different types of asphalt.

The density change curves of different kinds of asphalt models during the dynamic equilibrium of the NPT system at 298 K are shown in Figure 8.

It can be seen that during the kinetic simulation, the density change of the model reflects the gradual stabilization process of the internal configuration of the system. The density increased rapidly in the first 100 ps. This indicated that the components of the system were moving intensely and rearranging in the initial stage. Then, the intermolecular interactions were gradually strengthened, and the system tended to be more compactly arranged. Over time, the molecular movement tended to slow down. The structure gradually stabilized, the density changed, and it entered a smooth stage. This gradual stabilization of the density change indicated that the components reached equilibrium. The slow increase in density and the final oscillatory trend might be related to the minor local structural adjustments.

The mean density of the last 100 ps was selected to ensure the results were more representative. The density size relationship was calculated as aged base asphalt (1.021 g/cm^3^) > aged CRMA (1.006 g/cm^3^) > original base asphalt (1.003 g/cm^3^) > CRMA (0.990 g/cm^3^). Both the base asphalt and CRMA had higher densities after aging. This was due to the transformation of the asphalt fractions as the asphalt aged. A slight increase in the asphaltene content and a slight decrease in the aromatic, saturate, and resin content increased the density of the aged asphalt [42]. This resulted in a shift in the overall molecular weight distribution, forming a denser molecular structure and increasing the density of the asphalt. In addition, the elastic properties of rubber powder as a modifier may make the density of unaged CRMA relatively low. Mixing CR would increase the complexity of the system. However, it has an inhibitory effect on the movement of asphalt molecules, thus reducing the overall density. The aged CRMA model was constructed without considering the aging of CR. Therefore, density changes are mainly caused by asphalt aging. This results in the density of the aged CRMA being greater than that of the CRMA.

#### 3.2.2. Compatibility

The difference in *S_p_* can be used to characterize the compatibility between two polymeric materials. The smaller the difference in the *S_p_* of polymeric materials, the better the compatibility [43]. The optimized equilibrium model was selected to calculate the *S_p_*. The *S_p_* of asphalt and CR at 25 °C before and after aging and their differences were obtained, as shown in Table 3.

As seen from Table 3, the difference in *S_p_* between asphalt and CR after aging is about four times that before aging. This indicated that the compatibility of the two deteriorated after aging, and that the stability of the asphalt and rubber blend system deteriorated. According to the compatibility theory, the larger the difference in the relative molecular mass of the two polymers, the larger the difference in the free energy of the two polymers, and the more they do not dissolve well [42]. After aging, polymerization and oxidation occurred within the asphalt, and the light fraction was gradually transformed into the heavy fraction. The macromolecule content of asphalt increased, and the small molecule content decreased, so the compatibility between asphalt and CR macromolecules deteriorated after aging. The results provide a new technical idea for recycling aged CRMA. That is, respectively, using modified technology for aged CR and aged asphalt by narrowing the difference between the *S_p_* of the two to achieve miscible regeneration.

#### 3.2.3. Diffusion Properties

Characteristics such as the movement, adsorption, and diffusion of asphalt molecules on the aggregate surface are essential in influencing the properties of the asphalt–aggregate interface. Therefore, the mean square displacement (MSD) and diffusion coefficient were used to quantitatively characterize the diffusion motion of asphalt molecules on the aggregate surface.

The atoms in an MD calculation system constantly move from their starting positions, which are different at each instant. Thus, recording the trajectories of the molecules in a homogeneous orientation refers to the sum of the squares of the difference between the absolute value of the moving position *r*(*t*) and the initial position *r*(0) of all the particles in the system after *t* time. MSD can be used to determine whether the system is in equilibrium. Its value increases with the simulation time and stabilizes when all the particles in the system reach a balance, which is calculated as shown in Equation (5).

(5) $$MSD(t)=\frac{1}{N}{\sum_{i=1}^{N}\left[r_{i}\left(t\right)-r_{i}\left(0\right)\right]}^{2}$$

where *t* is the movement time; *r_i_*(*t*) is the position of particle *i* at time *t*; *r_i_*(0) is the position of particle *i* at time 0; and *N* is the number of molecules in the system.

According to the Einstein diffusion model, the molecular diffusion coefficient can be calculated as shown in Equation (6).

(6) $$D=\frac{1}{6}\underset{t\to \infty }{\lim}\frac{d}{dt}{\sum_{i=1}^{N}\left[r_{i}\left(t\right)-r_{i}\left(0\right)\right]}^{2}$$

where *D* is the molecular diffusion coefficient, *t* is the movement time, and *r_i_*(*t*) is the position of particle *i* at time *t*.

Then, the molecular diffusion coefficient can be obtained from the MSD as

(7) $$D=\frac{1}{6}\underset{t\to \infty }{\lim}\frac{dMSD}{dt}$$

##### Diffusion Properties of Asphalt Components

**Figure 9:**
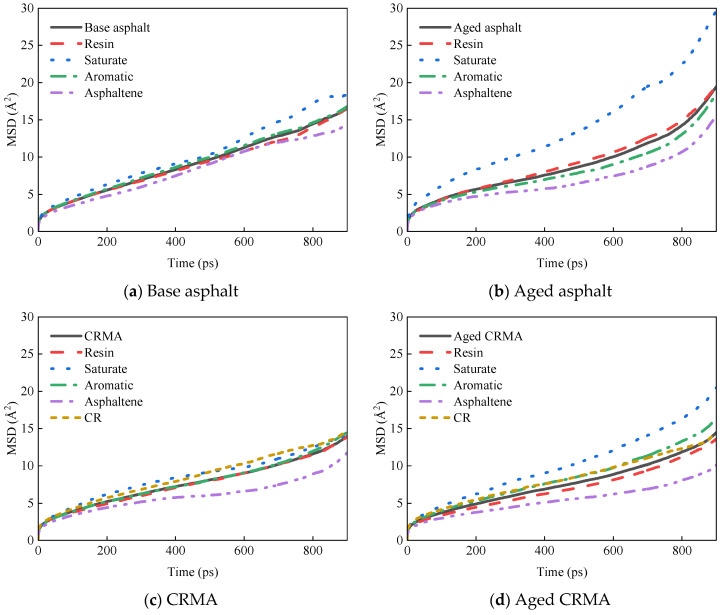
MSD curves of asphalt fractions under conditions of different asphalt types.

The optimized equilibrium state model was selected to analyze the MSD of each asphalt fraction. The sharp increase in MSD after 900 ps is due to the accumulation of computational errors, which can be eliminated from the analysis [44]. To better observe the MSD curve, the data from 0–900 ps were taken for plotting. The curves obtained for the MSD of asphalt and each fraction with simulation time are shown in Figure 9.

**Figure 10:**
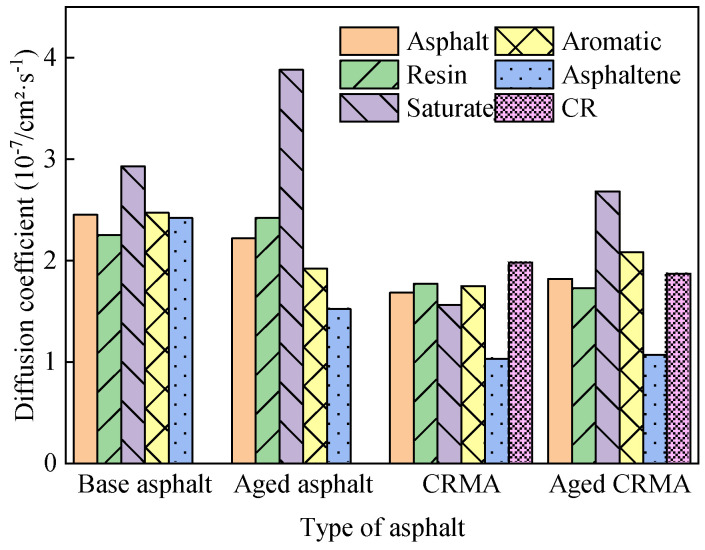
Diffusion coefficients of asphalt fractions.

**Figure 11:**
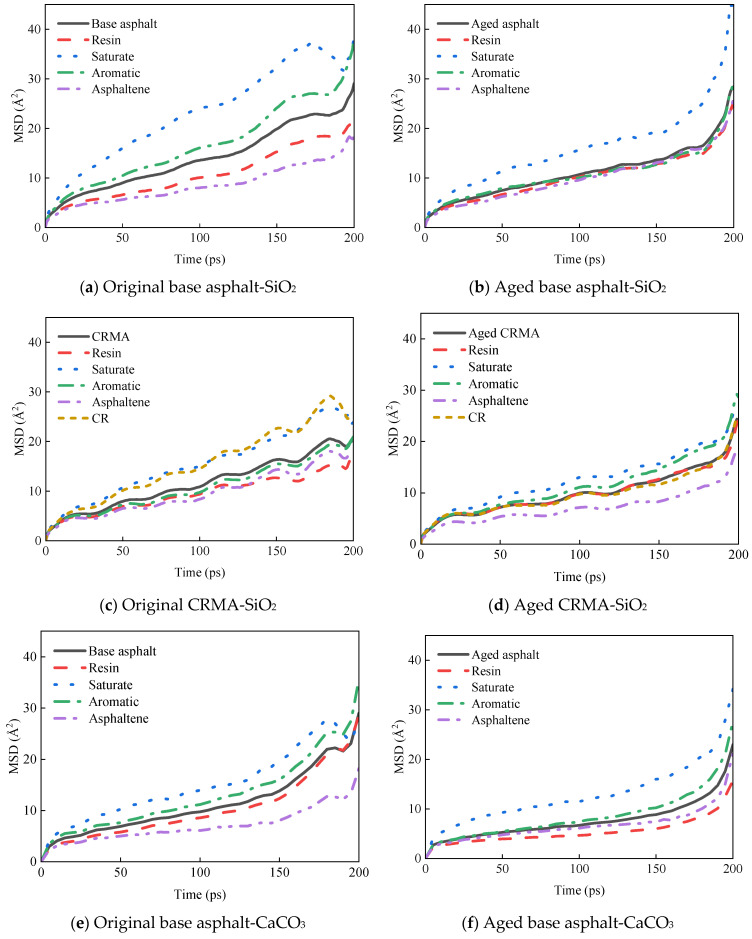

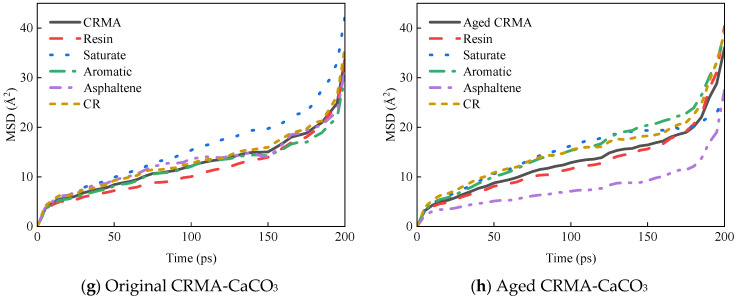
MSD curves of asphalt fractions moving on different aggregate surfaces.

Figure 10 shows that the MSD curves of all the fractions gradually increased as the simulation time grew, but there were differences in the growth rates of different fractions. Since the number of particles (*N*) was constant in the NPT ensemble, the slope between the MSD curve and *t* is proportional to the diffusion coefficient. The greater the slope of the MSD curve, the greater the diffusion coefficient and the greater the molecular diffusion ability. Calculating diffusion coefficients from MSD curves provides a better understanding of the diffusion properties of molecules. The data of the middle section of each MSD curve were selected for linear regression to obtain the slope of the curve, and the diffusion coefficient was calculated by combining it with Equation (7), as shown in Figure 10. The analysis in Figure 11 was as follows:

(1) Diffusion rates varied for each fraction of asphalt. The saturate fraction moved the fastest and the asphaltene the slowest, and the law holds for most asphalt species. This was mainly related to the relative molecular weights of the asphalt fractions, where molecules with large relative molecular weights moved more slowly, and molecules with small relative molecular weights moved more quickly [36]. They were less active because molecules with high relative molecular weights had larger molecular structures and higher bond-stretching energies. Molecules with small relative molecular weights had small changes in their bond angle energies and were more reactive [45].
(2) The addition of CR reduced the motion of asphalt molecules. The diffusion coefficient of CRMA decreased by about 31% compared to base asphalt, and the diffusion coefficients of the fractions of CRMA were also lower than those of base asphalt. Because the CR molecules were mixed with the fractions of the asphalt, the intermolecular interactions in the system inhibited the incorporation of CR, resulting in the slow molecular movement of CRMA. Therefore, CR would inhibit the movement of asphalt fractions and, to a certain extent, could improve the stability of CRMA. However, it was shown that there is a correlation between the asphalt diffusion coefficient and its self-healing properties. Smaller diffusion coefficients would weaken the self-healing performance of asphalt mixtures for microcracks [19].
(3) The diffusion coefficient of the base asphalt decreased by about 10% after aging. However, the diffusion coefficient of CRMA did not change much. Because the diffusion coefficient of asphalt fractions affected their rheological properties [36], the rheological properties of CRMA were more stable, and the aging resistance was better than that of base asphalt.

##### Diffusion Properties of Asphalt Components in Asphalt-Aggregate Interface Model

The MSD curves of each asphalt fraction on the aggregate surface are shown in Figure 11.

**Figure 12:**
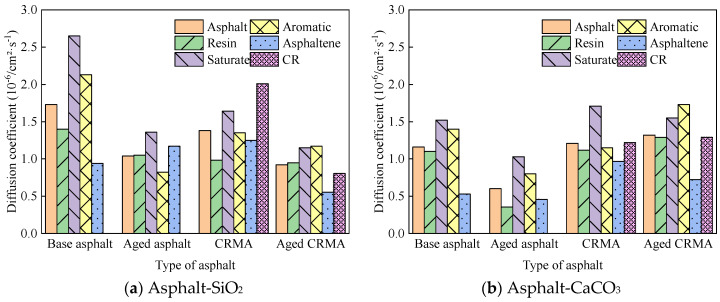
Diffusion coefficients of asphalt fractions moving on different aggregate surfaces.

The diffusion properties of molecules can be better understood by calculating the diffusion coefficients from MSD curves. The middle section of each MSD curve was selected for linear fitting to obtain the slope of the curve, and the diffusion coefficient was calculated in combination with Equation (7), as shown in Figure 12.

(1) The asphalt fractions of the asphalt–aggregate interface model had the fastest movement for the saturate fraction, followed by aromatic fraction and resin, and the slowest movement for asphaltene. The law of motion was consistent with the law mentioned above for the fractions of asphalt and held for most asphalt species. However, the diffusion coefficient of asphalt on the aggregate surface was nearly ten times greater than that of asphalt alone.
(2) Different aggregates affected the asphalt diffusion coefficient for the asphalt–aggregate interface. The diffusion coefficient of asphalt in the asphalt–SiO_2_ interface was greater than that of asphalt in the asphalt–CaCO_3_ interface, and this law held for most asphalt species.

### 3.3. Mechanical Properties

The bulk modulus reflects the ability of a material to resist external homogeneous compression in an elastic regime. The shear modulus reflects the ability of a material to resist shear deformation under shear stresses. Young’s modulus is a physical quantity that characterizes a material’s ability to resist deformation. In molecular simulations, any system subjected to an external force is in a state of stress, which causes a change in the relative positions of the particles in the system. For isotropic materials, the stress–strain behavior can be fully described by the Lamé constant alone. At this time, the stiffness matrix c of the system can be established by Lamé’s constant to establish its relationship with the stress–strain. Then, the bulk modulus K, shear modulus G, and Young’s modulus E of each system can be calculated as shown in Equations (8)–(13) [46]:

(8) $$K_{H}=\frac{K_{V}+K_{R}}{2}$$

(9) $$K_{V}=\frac{c_{11}+c_{22}+c_{33}+2(c_{12}+c_{23}+c_{13})}{9}$$

(10) $$K_{R}=\frac{1}{s_{11}+s_{22}+s_{33}+2(s_{12}+s_{23}+s_{13})}$$

(11) $$G_{H}=\frac{G_{V}+G_{R}}{2}$$

(12) $$G_{V}=\frac{c_{11}+c_{22}+c_{33}-(c_{12}+c_{23}+c_{31})+3(c_{44}+c_{55}+c_{66})}{15}$$

(13) $$G_{R}=\frac{15}{4(s_{11}+s_{22}+s_{33})-4(s_{12}+s_{23}+s_{13})+3(s_{44}+s_{55}+s_{66})}$$

where *K_V_*, *K_R_* and *K_H_* are the approximate upper limit, lower limit, and mean value of the bulk modulus of the Voigt–Reuss–Hill method, respectively; *G_V_*, *G_R_* and *G_H_* are the approximate upper limit, lower limit and mean value of the shear modulus of the Voigt–Reuss–Hill method, respectively; *c_ij_* (*i* = 1, 2, …, 6; *j* = 1, 2, …, 6) is the value of the components in the stiffness matrix; and *S_ij_* (*i* = 1, 2, …, 6; *j* = 1, 2, …, 6) is the value of the flexural matrix components.

The trajectory files of the last ten ps of the dynamic optimization process were selected for mechanical property analysis. The elastic stiffness matrix (GPa) and elastic flexibility matrix (1/TPa) can be obtained for each model. The following equations show the stiffness and flexibility matrix calculated from the base asphalt model:

$$c=\left[\begin{array}{cccccc} 2.6522 & 1.3818 & 1.7532 & -0.2537 & -0.1107 & 0.3025\\ 1.3818 & 2.7037 & 1.4184 & -0.1575 & -0.0013 & 0.0120\\ 1.7532 & 1.4184 & 3.2986 & -0.2243 & 0.0694 & -0.1205\\ -0.2537 & -0.1575 & -0.2243 & 0.7069 & -0.0353 & 0.0024\\ -0.1107 & -0.0013 & 0.0694 & -0.0353 & 0.8911 & -0.0855\\ 0.3025 & 0.0120 & -0.1205 & 0.0024 & -0.0855 & 0.7624 \end{array}\right]$$

$$S=\left[\begin{array}{cccccc} 745.8934 & -208.4951 & -312.1299 & 127.8754 & 89.8589 & -332.3464\\ -208.4951 & 538.5181 & -118.0670 & 6.9557 & -10.4446 & 54.3662\\ -312.1299 & -118.0670 & 530.2796 & 26.2528 & -59.7428 & 202.7558\\ 127.8754 & 6.9557 & 26.2528 & 1474.0505 & 68.0802 & -43.7919\\ 89.8589 & -10.4446 & -59.7428 & 68.0802 & 1148.7073 & 83.6310\\ -332.3464 & 54.3662 & 202.7558 & -43.7919 & 83.6310 & 1484.2970 \end{array}\right]$$

**Figure 13:**
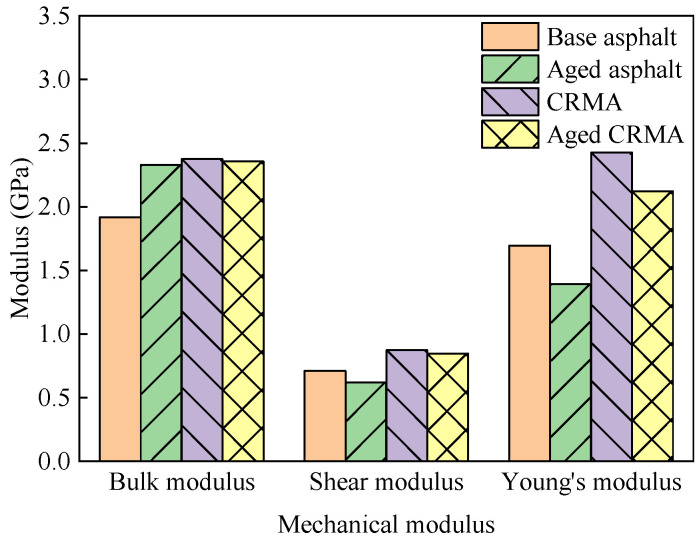
Variation in the modulus of different types of asphalt.

Each model’s stiffness and flexibility matrices were analyzed using the abovementioned equations. The bulk modulus *K* with shear modulus *G* and Young’s modulus *E* were obtained for each model, respectively, and the calculated results are shown in Figure 13.

(1) Compared with base asphalt, CRMA increased each mechanical modulus. This included an increase in the bulk modulus of about 24%, shear modulus of about 23%, and Young’s modulus of about 43%. This indicated that CR improved the mechanical properties of asphalt and, through interaction with asphalt fractions, inhibited the movement of asphalt fractions.
(2) The bulk modulus of the base asphalt increased by 21% after aging. The higher the bulk modulus, the smaller the material’s volume change and the greater the resistance to compressive force. This may be due to polymerization and oxidation within the asphalt after aging. The light fractions of asphalt are gradually converted to heavy fractions, so the asphalt becomes brittle and hard after aging. The shear modulus and Young’s modulus of base asphalt and CRMA decreased after aging. The shear modulus of the aged asphalt decreased by about 13% and Young’s modulus decreased by about 18%. The shear modulus of the aged CRMA decreased by about 3% and Young’s modulus decreased by about 13%. This indicated that the mechanical properties of both base asphalt and CRMA deteriorated after aging. However, CRMA showed a smaller reduction than base asphalt. This indicated that CRMA has better aging resistance.

### 3.4. Component Interaction Behavior

#### 3.4.1. Energy Analysis

**Figure 14:**
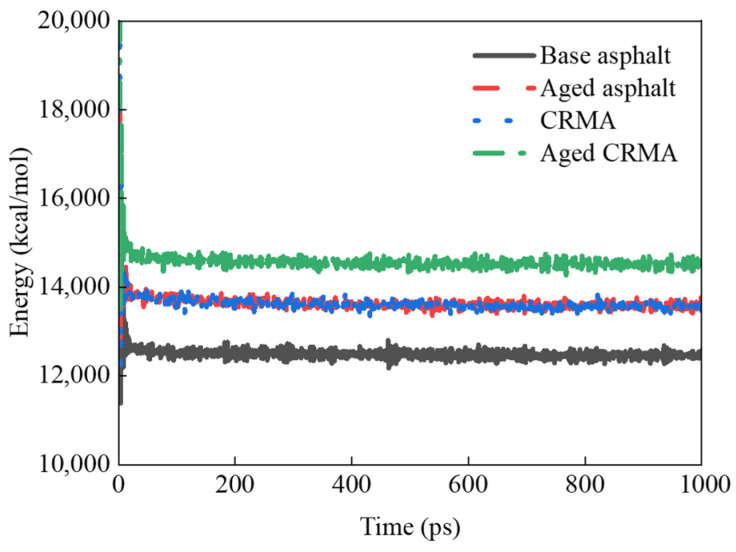
Energy variation for different asphalts.

The energy change curves during the dynamic equilibrium of NPT system synthesis at 298 K for different kinds of asphalt models are shown in Figure 14.

During the kinetic simulation, the model’s energy fluctuated greatly in the first 100 ps and stabilized as the iteration time increased. The last 100 ps energy average was taken as the representative energy. The following results were obtained:

(1) The energy size relationship of various types of asphalt was calculated as aged CRMA (14,507 kcal/mol) > aged asphalt (13,575 kcal/mol) > CRMA (13,564 kcal/mol) > base asphalt (12,465 kcal/mol). CRMA provided an 8.8% total energy gain over base asphalt. Because CR is a macromolecule, adding it to asphalt increases its energy.
(2) The total energy of base asphalt and CRMA was improved by 8.9% and 6.9% after aging, respectively. The molecular mass of asphalt increased due to the oxidation of small molecules to form large molecules of asphaltene during the aging process. According to the mass–energy equation, an increase in mass at a given speed would increase the energy of the substance. This resulted in a greater relative atomic mass and higher energy in the aged asphalt than in the base asphalt [42].

#### 3.4.2. Interaction Energy Analysis

**Figure 15:**
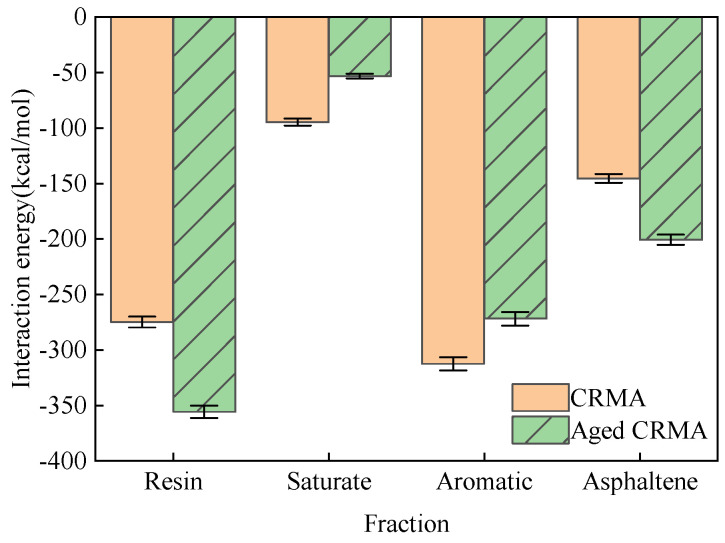
Interaction energy of asphalt fractions with CR before and after aging.

Interaction energy is an important index for evaluating the strength of the interaction between two substances. When its value is negative, it indicates gravitational force. The larger the absolute value, the stronger the force. The last 100 ps trajectory file during the kinetic simulation was selected, and the interaction energy averages were calculated. The interaction energies of the asphalt fractions with the CR before and after aging were obtained, as shown in Figure 15.

(1) In CRMA, the absolute value of the interaction energy between CR and each fraction was calculated as aromatic > resin > asphaltene > saturate. Considering that the content of the four fractions in the asphalt was not uniform, the interaction energy was divided by the mass percentage of the four fractions (Table 1) to make the simulation results more instructive. The magnitude relationship of the normalized fitting results was obtained as aromatic (−976.98 kcal/mol) > saturate (−853.38 kcal/mol) > asphaltene (−840.98 kcal/mol) > resin (−694.42 kcal/mol). The interaction energy of light fractions such as saturate and aromatic with CR was stronger, indicating that CR will physically adsorb with light fractions.
(2) In aged CRMA, the absolute value of the interaction energy between CR and each fraction was calculated as resin > aromatic > asphaltene > saturate. The magnitude relationship of the normalized fitting results was obtained as resin (−964.07 kcal/mol) > aromatic (−927.88 kcal/mol) > asphaltene (−832.68 kcal/mol) > saturate (−853.38 kcal/mol). The interaction energy between saturates and CR became weaker, and the interaction energy between resin and CR became stronger after aging.

### 3.5. Asphalt–Aggregate Interface Interaction

Interaction energy is an important index for evaluating the strength of the interaction between two substances. When its value is negative, it indicates gravitational force. The larger the absolute value is, the stronger the force is, and its calculation formula is shown in Equation (14).

(14) $$E_{\mathrm{inter}}=E_{A-B}-(E_{A}+E_{B})$$

where *E_inter_* (kcal/mol) is the interaction energy between molecules; *E_A−B_* (kcal/mol) is the total energy after equilibrium in the mixed system; *E_A_* (kcal/mol) is the total energy after the equilibrium of material *A* in the mixed system; and *E_B_* (kcal/mol) is the total energy after the equilibrium of material *B* in the mixed system.

For the asphalt–aggregate interface model, the work of adhesion can be defined as the work performed per unit area when separating the interface between the two phases to generate two free surfaces, which is calculated as shown in Equation (15).

(15) $$W_{adhesion}=\frac{\Delta E}{A}\times K=\frac{-E_{\mathrm{inter}}}{A}\times K$$

where *W_adhesion_* (mJ·m^2^) is the adhesion work at the interface; Δ*E* (kcal/mol) is the binding energy between the asphalt and aggregate, i.e., the negative value of the interaction energy; and *A* (Å^2^) is the cross-sectional area of the interface. Conversion factor unit *K* = 695.

**Figure 16:**
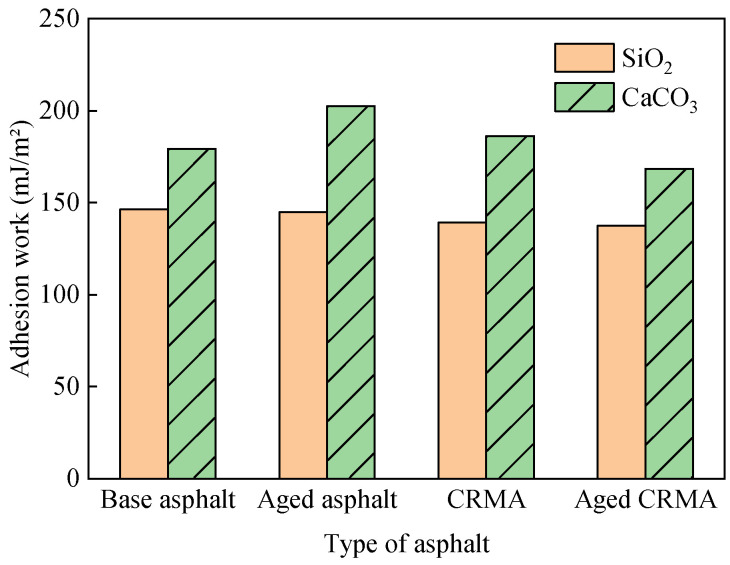
Adhesion work of asphalt–aggregate.

The 100 ps trajectory file after the kinetic equilibrium process was selected to calculate the mean interaction energy. The adhesion work was calculated according to Equation (15), as shown in Figure 16.

(1) The asphalt–aggregate interfacial adhesion work was affected by the aggregate type. The adhesion work of CaCO_3_ with asphalt was greater than that of SiO_2_. The difference between the adhesion work of the two aggregates with different asphalts was in the range of 22.5–39.9%. Because SiO_2_ was an acidic aggregate, and CaCO_3_ was a basic aggregate, acidic aggregates had worse adhesion properties with asphalt [47]. It can also be seen that the adhesion work diffusion law was consistent with the diffusion law of asphalt and its fractions on different aggregate surfaces. Aggregates with strong adherence were slower to diffuse. Aggregates with less adhesion work spread faster.
(2) After adding CR, the asphalt–SiO_2_ interfacial adhesion work decreased by 5.2%, and the asphalt–CaCO_3_ interfacial adhesion work did not change much. This indicated that adding CR reduced the adhesion properties at the asphalt–aggregate interface. It should be emphasized that this conclusion presupposes that the mass of base asphalt in CRMA was equal to the modeled mass of base asphalt in the asphalt–aggregate adhesion model. This also indirectly verified that the optimal oil/stone ratio of CRMA mixes was larger than that of base asphalt mixes when designing CRMA mix ratios [48].

## 4. Conclusions

In this study, the modification, aging, and interfacial interaction with aggregates mechanisms of CRMA were investigated by molecular dynamics. Different asphalt and asphalt-aggregate interface molecular models were constructed. The mechanisms were analyzed using density, *S_p_*, MSD, diffusion coefficient, mechanical modulus, and adhesion work. The main conclusions can be drawn as follows:

(1) Compared to the base asphalt, the mechanical modulus of CRMA increased by 23–43%, and the diffusion coefficient decreased by about 31%. This indicated that the CR enhanced the interactions between the asphalt systems, improving the mechanical properties. The CR inhibited the movement of the asphalt components (especially saturate and aromatic) through interactions with the asphalt components.
(2) The *S_p_* difference between aged asphalt and CR was about four times that before aging. It indicated that the compatibility between asphalt and CR deteriorated obviously after aging. Therefore, compatibility should be a key concern when rubberized asphalt is recycled.
(3) The shear modulus and Young’s modulus of both base asphalt and CRMA decreased after aging. The decrease for CRMA was smaller than that of base asphalt. The diffusion coefficient of the base asphalt decreased by about 10% after aging, but the CRMA remained stable. This indicated that CR could improve the mechanical stability of the base asphalt. This is attributed to the CR inhibiting the diffusion of asphalt components, providing CRMA with better aging resistance.
(4) The adhesion work of CaCO_3_ with asphalt was greater than that of SiO_2_, and the difference between the adhesion work of the two aggregates with different asphalts was 22.5–39.9%. It can be seen that the diffusion law of adhesion work was consistent with the diffusion law of asphalt. Aggregates with stronger adhesion had slower diffusion rates. Aggregates with less adhesion had a faster diffusion rate.

The research results of this paper provide good theoretical guidance for the application of CRMA. However, there are some simplifications in the modeling process. This study did not consider the solubilization of CR in asphalt. The effects of aggregate mineral surface anisotropy were not considered in the modeling process. Therefore, the above issues could be further studied in the future.

## Figures and Tables

**Table 1:** Molecular composition of the base and aged asphalt model.

Fraction	Molecular	Base Asphalt			Aged Asphalt		
		Molecular Formula	Number of Molecules	Fraction Content (%)	Molecular Formula	Number of Molecules	Fraction Content (%)
Resin	Pyridinohopane	C_36_H_57_N	4	39.6	C_36_H_57_N	3	36.9
	Thio-isorenieratane	C_40_H_60_S	4		C_40_H_60_SO	4	
	Trimethylbenzene-oxane	C_29_H_50_O	5		C_29_H_50_O	4	
	Quinolinohopane	C_40_H_59_N	4		C_40_H_59_N	3	
	Benzobisbenzothiophene	C_18_H_10_S_2_	15		C_18_H_10_S_2_O_2_	15	
Saturate	Squalane	C_30_H_62_	4	11.1	C_30_H_62_	4	9.7
	Hopane	C_35_H_62_	4		C_35_H_62_	3	
Aromatic	PHPN	C_35_H_44_	11	32.0	C_35_H_44_	10	29.3
	DOCHN	C_30_H_46_	13		C_30_H_46_	12	
Asphaltene	Pyrrole	C_66_H_81_N	2	17.3	C_66_H_81_N	3	24.1
	Phenol	C_42_H_54_O	3		C_42_H_54_O	4	
	Thiophene	C_51_H_62_S	3		C_51_H_62_SO	4	

**Table 2:** Repeat unit content of each structure of SBR (ω/%).

Styrene	Trans-1,4-Butadiene	Cis-1,4-Butadiene	1,2-Butadiene	Others
23.5	58	5.5	12	1

**Table 3:** *S_p_* of asphalt and CR and their differences before and after aging at 25 °C.

Parameter	CR	Original Base Asphalt	Aged Base Asphalt
*S_p_*/[(J/m^−3^)^1/2^]	18.020	18.214	18.753
|Δ*S_p_*|/[(J/m^−3^)^1/2^]	/	0.194	0.733

## Data Availability

All data, models, and code generated or used during the study appear in the published article.

## References

[1] Presti D.L. (2013). Recycled Tyre Rubber Modified Bitumens for road asphalt mixtures: A literature review. Constr. Build. Mater..

[2] Poovaneshvaran S., Hasan M.R.M., Jaya R.P. (2020). Impacts of recycled crumb rubber powder and natural rubber latex on the modified asphalt rheological behaviour, bonding, and resistance to shear. Constr. Build. Mater..

[3] Shisong R., Xueyan L., Jian X., Peng L. (2021). Investigating the role of swelling-degradation degree of crumb rubber on CR/SBS modified porous asphalt binder and mixture. Constr. Build. Mater..

[4] Carlo C., Edoardo B., Emiliano P., Maurizio B. (2022). Evaluation of the rheological and performance behaviour of bitumen modified with compounds including crumb rubber from waste tires. Constr. Build. Mater..

[5] Wang T., Xiao F., Zhu X., Huang B., Wang J., Amirkhanian S. (2018). Energy consumption and environmental impact of rubberized asphalt pavement. J. Clean. Prod..

[6] Bakar S.K.A., Abdullah M.E., Kamal M.M., Rahman R.A., Buhari R., Jaya R.P., Sabri S., Ahmad K.A. (2018). The effect of crumb rubber on the physical and rheological properties of modified binder. J. Phys. Conf. Ser..

[7] Wenhui Z., Xiangbing X., Guanghui L., Jiuguang G., Meng B., Mingwei W. (2020). Research on the Influence of Nanocarbon/Copolymer SBS/Rubber Powder Composite Modification on the Properties of Asphalt and Mixtures. Adv. Mater. Sci. Eng..

[8] Tang N., Huang W., Hu J., Xiao F. (2018). Rheological characterisation of terminal blend rubberised asphalt binder containing polymeric additive and sulphur. Road Mater. Pavement Des..

[9] Peng W., Li P., Gao J., Liu Z., Wang X., Wang S., Wu W. (2024). Long-term skid resistance evolution and influence mechanism of asphalt pavement based on self-developed wear equipment. Constr. Build. Mater..

[10] Jeong K., Lee S., Amirkhanian S.N., Kim K.W. (2010). Interaction effects of crumb rubber modified asphalt binders. Constr. Build. Mater..

[11] Peng W., Li P., Gong W., Tian S., Wang Z., Liu S., Liu Z. (2023). Preparation and mechanism of rubber-plastic alloy crumb rubber modified asphalt with low viscosity and stabilized performance. Constr. Build. Mater..

[12] Su J., Li P., Zhu G., Wang X., Dong S. (2024). Interface Interaction of Waste Rubber—Asphalt System. Buildings.

[13] Wang H., You Z., Mills-Beale J., Hao P. (2011). Laboratory evaluation on high temperature viscosity and low temperature stiffness of asphalt binder with high percent scrap tire rubber. Constr. Build. Mater..

[14] Chen Z., Wang T., Pei J., Amirkhanian S., Xiao F., Ye Q., Fan Z. (2019). Low Temperature and Fatigue Characteristics of Treated Crumb Rubber Modified Asphalt after a Long Term Aging Procedure. J. Clean. Prod..

[15] Shen A., Zhai C., Guo Y., Yang X. (2018). Mechanism of adhesion property between steel slag aggregate and rubber asphalt. J. Adhes. Sci. Technol..

[16] Zhou X., Moghaddam T.B., Chen M., Wu S., Adhikari S., Wang F., Fan Z. (2021). Nano-scale analysis of moisture diffusion in asphalt-aggregate interface using molecular simulations. Constr. Build. Mater..

[17] Zheng C., Shan C., Liu J., Zhang T., Yang X., Lv D. (2021). Microscopic adhesion properties of asphalt-mineral aggregate interface in cold area based on molecular simulation technology. Constr. Build. Mater..

[18] Sonibare K., Rucker G., Zhang L. (2021). Molecular dynamics simulation on vegetable oil modified model asphalt. Constr. Build. Mater..

[19] Hu D., Pei J., Li R., Zhang J., Jia Y., Fan Z. (2020). Using thermodynamic parameters to study self-healing and interface properties of crumb rubber modified asphalt based on molecular dynamics simulation. Front. Struct. Civ. Eng..

[20] Xu G., Wang H. (2017). Molecular dynamics study of oxidative aging effect on asphalt binder properties. Fuel.

[21] Wang L., Liu Y., Zhang L. (2020). Micro/Nanoscale Study on the Effect of Aging on the Performance of Crumb Rubber Modified Asphalt. Math. Probl. Eng..

[22] Qu X., Liu Q., Guo M., Wang D., Oeser M. (2018). Study on the effect of aging on physical properties of asphalt binder from a microscale perspective. Constr. Build. Mater..

[23] Chen P., Luo X., Gao Y., Zhang Y. (2022). Modeling percentages of cohesive and adhesive debonding in bitumen-aggregate interfaces using molecular dynamics approaches. Appl. Surf. Sci..

[24] Gong Y., Xu J., Yan E. (2021). Intrinsic temperature and moisture sensitive adhesion characters of asphalt-aggregate interface based on molecular dynamics simulations. Constr. Build. Mater..

[25] Li D.D., Greenfield M.L. (2014). Chemical compositions of improved model asphalt systems for molecular simulations. Fuel.

[26] Xu Z., Wang Y., Cao J., Chai J., Cao C., Si Z., Li Y. (2021). Adhesion between asphalt molecules and acid aggregates under extreme temperature: A ReaxFF reactive molecular dynamics study. Constr. Build. Mater..

[27] Chen W., Chen S., Zheng C. (2021). Analysis of micromechanical properties of algae bio-based bio-asphalt-mineral interface based on molecular simulation technology. Constr. Build. Mater..

[28] Li M., Liu L., Xing C., Liu L., Wang H. (2021). Influence of rejuvenator preheating temperature and recycled mixture’s curing time on performance of hot recycled mixtures. Constr. Build. Mater..

[29] He L., Zheng Y., Alexiadis A., Cannone Falchetto A., Li G., Valentin J., Van den Bergh W., Emmanuilovich Vasiliev Y., Kowalski K.J., Grenfell J. (2021). Research on the self-healing behavior of asphalt mixed with healing agents based on molecular dynamics method. Constr. Build. Mater..

[30] Zhang L., Long N., Liu Y., Wang L. (2022). Cross-scale study on the influence of moisture-temperature coupling conditions on adhesive properties of rubberized asphalt and steel slag. Constr. Build. Mater..

[31] Wang L., Zhang L., Liu Y. (2022). Molecular Dynamics Study on the Effect of Mineral Composition on the Interface Interaction between Rubberized Asphalt and Aggregate. J. Mater. Civ. Eng..

[32] Jiao B., Pan B., Che T. (2022). Evaluating impacts of desulfurization and depolymerization on thermodynamics properties of crumb rubber modified asphalt through molecular dynamics simulation. Constr. Build. Mater..

[33] Guo F., Zhang J., Pei J., Ma W., Hu Z., Guan Y. (2020). Evaluation of the compatibility between rubber and asphalt based on molecular dynamics simulation. Front. Struct. Civ. Eng..

[34] Guo F., Zhang J., Pei J., Zhou B., Falchetto A.C., Hu Z. (2020). Investigating the interaction behavior between asphalt binder and rubber in rubber asphalt by molecular dynamics simulation. Constr. Build. Mater..

[35] Zhang X., Han C., Otto F., Zhang F. (2021). Evaluation of Properties and Mechanisms of Waste Plastic/Rubber-Modified Asphalt. Coatings.

[36] Khabaz F., Khare R. (2015). Glass Transition and Molecular Mobility in Styrene-Butadiene Rubber Modified Asphalt. J. Phys. Chem. B.

[37] Guo F., Pei J., Zhang J., Xue B., Sun G., Li R. (2020). Study on the adhesion property between asphalt binder and aggregate: A state-of-the-art review. Constr. Build. Mater..

[38] Lesueur D. (2008). The colloidal structure of bitumen: Consequences on the rheology and on the mechanisms of bitumen modification. Adv. Colloid Interface Sci..

[39] You L., Spyriouni T., Dai Q., You Z., Khanal A. (2020). Experimental and molecular dynamics simulation study on thermal, transport, and rheological properties of asphalt. Constr. Build. Mater..

[40] Zhou K., Huang J., Deng Y., Huang L. (2021). Molecular dynamics simulation of the interface mechanical properties of graphene modified asphalt. J. Funct. Mater..

[41] Wang P., Dong Z., Tan Y., Liu Z. (2015). Investigating the Interactions of the Saturate, Aromatic, Resin, and Asphaltene Four Fractions in Asphalt Binders by Molecular Simulations. Energy Fuels.

[42] Wang L., Zhang L., Liu Y. (2019). Molecular Dynamics Study on Compatibility of Asphalt and Rubber Powders before and after Aging. J. Build. Mater..

[43] Wang L., Zhang L., Liu Y. (2018). Compatibility of Rubber Powder and Asphalt in Rubber Powder Modified Asphalt by Molecular Dynamics. J. Build. Mater..

[44] Hu D., Gu X., Cui B. (2021). Effect of styrene-butadiene-styrene copolymer on the aging resistance of asphalt: An atomistic understanding from reactive molecular dynamics simulations. Front. Struct. Civ. Eng..

[45] Gao Y., Xie Y., Liao M., Li Y., Zhu J., Tian W. (2023). Study on the mechanism of the effect of graphene on the rheological properties of rubber-modified asphalt based on size effect. Constr. Build. Mater..

[46] Su M., Zhang H., Zhang Y., Zhang Z. (2017). Miscibility and mechanical properties of SBS and asphalt blends based on molecular dynamics simulation. J. Chang’an Univ. (Nat. Sci. Ed.).

[47] Yao H., Liu J., Xu M., Ji J., Dai Q., You Z. (2022). Discussion on molecular dynamics (MD) simulations of the asphalt materials. Adv. Colloid Interface Sci..

[48] Kim J.R. (2001). Characteristics of crumb rubber modified (CRM) asphalt concrete. KSCE J. Civ. Eng..

